# Microencapsulation of Purified Amylase Enzyme from Pitaya (*Hylocereus polyrhizus*) Peel in Arabic Gum-Chitosan using Freeze Drying

**DOI:** 10.3390/molecules19033731

**Published:** 2014-03-24

**Authors:** Mehrnoush Amid, Yazid Manap, Nor Khanani Zohdi

**Affiliations:** Department of Food Technology, Faculty of Food Science and Technology, Universiti Putra Malaysia, 43400 UPM Serdang, Selangor 43400, Malaysia; E-Mails: myazid@upm.edu.my (Y.M.); norkhanani@gmail.com (N.K.Z.)

**Keywords:** microencapsulation, freeze-drying, scanning electron microscope, amylase, chitosan, Arabic gum, stability, efficiency

## Abstract

Amylase is one of the most important enzymes in the world due to its wide application in various industries and biotechnological processes. In this study, amylase enzyme from *Hylocereus polyrhizus* was encapsulated for the first time in an Arabic gum-chitosan matrix using freeze drying. The encapsulated amylase retained complete biocatalytic activity and exhibited a shift in the optimum temperature and considerable increase in the pH and temperature stabilities compared to the free enzyme. Encapsulation of the enzyme protected the activity in the presence of ionic and non-ionic surfactants and oxidizing agents (H_2_O_2_) and enhanced the shelf life. The storage stability of amylase is found to markedly increase after immobilization and the freeze dried amylase exhibited maximum encapsulation efficiency value (96.2%) after the encapsulation process. Therefore, the present study demonstrated that the encapsulation of the enzyme in a coating agent using freeze drying is an efficient method to keep the enzyme active and stable until required in industry.

## 1. Introduction

Amylases are enzymes that catalyze the initial hydrolysis of starch into shorter oligosaccharides, an important step towards transforming starch into single sugar units [1,2]. This class of enzyme holds the maximum market share of enzyme sales with its major application in the food industry [3]. With the advent of new frontiers in biotechnology, the spectrum of amylase application has also expanded to automatic dishwashing detergents as well as textile desizing and the pulp and paper industry [4]. It is also used in the pharmaceutical industry as a digestive aid [3]. *Hylocereus polyrhizus*, known as pitaya fruit, is one of the important commercial tropical fruits in the world because of its economic value and potential health benefits. About 33% of whole fruit weight is in the pitaya peel [5], which is often discarded as waste during processing, especially in the beverage production industries, or used as animal feed. However, there are different types of enzymes in the peel and it can be used as a rich and cost effective source for commercial production of natural and valuable kinds of enzymes such as amylase. Alteration or destruction of the natural morphology of the purified enzyme, which causes a decrease in enzyme activity and stability, could occur during storage time until required in industry. One of the most important drying processes for the conservation of enzymes and bioactive products is freeze-drying [6]. This technique can produce a fine, homogeneous powder with excellent control over impurity levels and a decrease in its environmental impact [7]. However, this method of drying generates different types of stresses, e.g., low temperature stress, dehydration stress and ice crystal formation, which deactivate and destabilize the enzymes [8]. Thus, many kinds of stabilizers have been used to decrease the deactivation and destabilization of freeze-dried enzymes [9]. Gum Arabic, with its solubility, low viscosity, emulsification qualities and excellent retention of volatile compounds, is very versatile for purposes of encapsulation.

Gum Arabic is able to form a dried matrix around dispersed compounds during dehydration processes, which entraps them inside the matrix and prevents volatile loss and contact with air. These solubility and surface-active qualities have facilitated its extensive use as an encapsulation matrix for retention and protection of enzymes [10,11]. In addition, chitosan exhibits many interesting properties, namely biocompatibility, availability of reactive functional groups for chemical modifications and mechanical stability. Furthermore, chitosan has been extensively used to encapsulate active substances in various industries due to its lack of toxicity, its film forming capacity and tensile strength [12]. It should be noted that, chitosan has a synergistic effect with Arabic gum, thus, because of the unique characteristics of both coating agents, as mentioned above and also their interactive properties, Arabic gum and chitosan were selected as an effective wall material for encapsulation of the amylase from *Hylocereus polyrhizus*. The encapsulation of amylase in combination with chitosan and Arabic gum using freeze drying has not been reported to-date. Therefore, in the present study, a combination of Arabic gum and chitosan was used for the first time along with freeze drying for the encapsulation of amylase to keep it in a highly active and stable state for potential application in various industries. In addition, the effects of encapsulation on the stability of the amylase in the presence of surfactant and oxidizing agent, as well as temperature stability, pH stability, encapsulation efficiency and storage stability of the encapsulated amylase were investigated in the study.

## 2. Result and Discussion

### 2.1. Efficiency of Encapsulated Amylase

Encapsulation efficiency is defined as the ratio of enzyme activity after encapsulation to the enzyme activity before encapsulation multiplied 100 fold:

Encapsulation efficiency (%) = Enzyme activity after encapsulation/initial enzyme activity × 100
(1)

**Figure 1:**
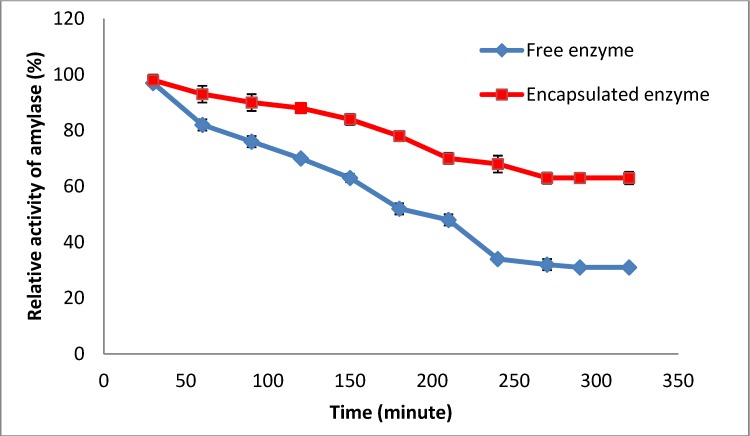
Effect of encapsulation on the amylase efficiency.

Encapsulation of the amylase in Arabic gum and chitosan using freeze drying protects the encapsulated enzyme’s activity and hence the stability of the encapsulated amylase. A similar result has been reported by DeGroot and Neufled [13] who indicated the encapsulation of urease in alginate and chitosan enhanced the enzyme activity. In is obviously important that the enzyme maintains its activity during encapsulation. One of the factors that govern enzyme activity is its water activity and reactivation of an enzyme can be achieved by increasing the water activity upon water uptake [14]. In an encapsulation controlled release system the most important parameter is equilibration with water upon water uptake. Encapsulation of enzyme in Arabic gum provided an example of a swelling-controlled system for controlled release, whereby the gum as coating agent protects the enzyme activity by controlling the release of enzyme [15]. The encapsulation efficiency was determined by investigation of the activity of free and encapsulated enzyme within 5 h and 30 min (with intervals of 30 min). Since the encapsulation efficiency of an enzyme has a direct correlation with the activity of the encapsulated enzyme, thus, increasing of enzyme activity of encapsulated enzyme leads to an increase in enzyme efficiency [16]. Based on the results shown in Figure 1, the residual enzyme activity of enzyme encapsulated in Arabic gum and chitosan was higher than that of the free enzyme. Thus, demonstrating that chitosan and Arabic gum caused an increase in the amylase activity due to the increased activity of the enzyme. This seems to be related to a formation of a suitable enzyme support complex that accesses the substrate without restriction. An encapsulation efficiency of 96.2% ± 1.1% of the encapsulated amylase was achieved after freeze drying of the enzyme in the matrix of Arabic gum-chitosan as coating agent.

### 2.2. Effect of Enzyme Encapsulation on pH Stability

**Figure 2:**
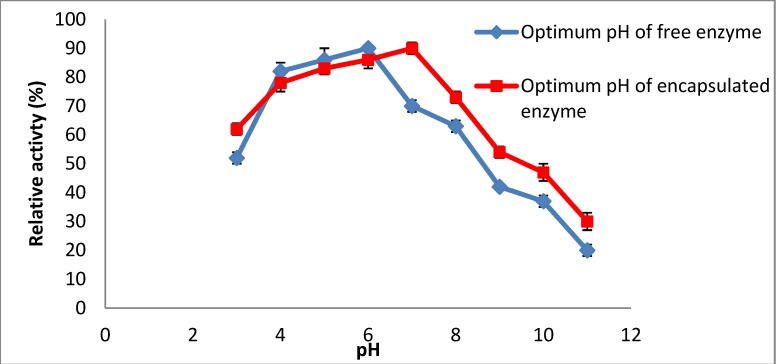
Optimum enzyme activity over wide range of pH.

The effect of pH on the catalytic activities of free and encapsulated amylase was investigated by determining the relative activities at different pH values. The activities of encapsulated and native enzymes were estimated at different pHs. A graph of relative activity *versus* pH was produced (Figure 2). The result indicated that the enzyme is more stable at acidic pH (pH 6.0) but encapsulated amylase showed the highest activity at pH 7.0 (Figure 1). The difference between the optimum pH for free and encapsulated amylase was about 1 unit. A similar observation was reported by Alemzadeh and Nejati [17] who also stated that the optimum pH of immobilized enzyme was shifted up 1.1 unit compared to the free enzyme. The difference could be due to the interior microenvironment of the microcapsule, which is cationic and separated from the membrane that is ionic in nature. There is another reason for this phenomenon which is the high concentration of hydrogen ion (H^+^) in the encapsulated amylase in comparison with the ion around the encapsulated amylase. Hence, to achieve a balanced concentration of ions in the two environments, hydrogen ions (H^+^) should be released from the inside to the outside of the environment [17], thus, the optimum pH is shifted from acidic to neutral.

### 2.3. Effect of Enzyme Encapsulation on Temperature Stability

**Figure 3:**
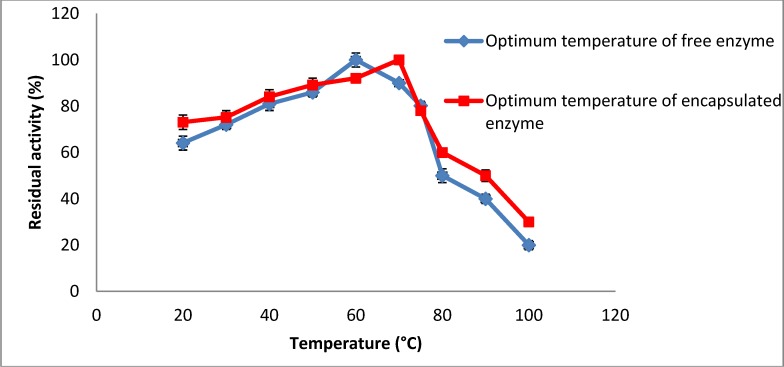
Effect of temperature on enzyme residual activity.

The enzymatic activity is dependent on the temperature in the same way as chemical catalysts, except that there is an optimum temperature for the enzymatic reaction above which the activity decreases due to denaturation of the enzyme protein. The activity of the immobilized amylase was measured using a UV spectrophotometer and using DNS as the substrate. The maximum activity of the immobilized enzyme is obtained at about 70 °C (Figure 3) compared to around 60 °C for the free enzyme. Thus the enzyme immobilization using freeze drying increased the temperature tolerance of the enzyme in the presence of coating agents. The enhancement in optimum temperature of the enzyme may be due to a diffusional effect, that is a greater diffusion flux through the chitosan and Arabic gum, and thus, a more adequate environment for the action of the enzyme with substrates. A similar observation was reported by Zhu and Chen [18] who immobilized β-galactosidase from *Kluveromyces lactis*.

### 2.4. Storage Stability of Encapsulated Amylase

One of the limiting factors for the application of enzymes in the food industry is inactivation of the enzyme during storage. It should be noted that enzymes require a certain level of water in their structures in order to maintain their natural conformation during storage, allowing them to deliver their full functionality after storage [19]. Thus, the water activity of the encapsulated amylase is one of the important factors to keep the enzyme active and stable during storage time and it can be used as a tool to inhibit the enzyme activity during storage and increase the shelf life of the enzyme [20]. It should be mentioned that water activities of the encapsulated and free enzyme were 92% and 83%, respectively. The combination of Arabic gum and chitosan and freeze drying make a rigid matrix which is able to control the water mobility of the enzyme during storage and enhance the storage stability of the enzyme. In addition, the mixture of Arabic gum and chitosan protects the amylase surface from possible oxidation and degradation during the storage procedure. It should be explained that the immobilization of the enzyme in the matrix (*i.e.*, combination of Arabic gum and chitosan) enhances the density of immobilized enzyme to improve its storage stability.

**Figure 4:**
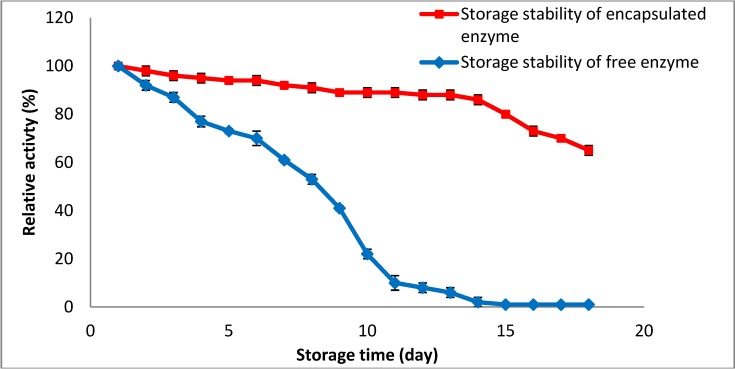
Effect of storage stability on enzyme residual activity.

Since the storage stability of the amylase has a direct correlation with enzyme activity, an increase in the enzyme activity after encapsulation leads to an increase in the storage stability of the amylase [21]. Therefore, based on the results as shown in Figure 4, the activity of the encapsulated amylase could be well maintained for up to 14 days. It means that 90.3% ± 0.01% of the enzyme activity was retained after storage of the encapsulated amylase at 4 °C, proving that the coating agent for enzyme encapsulation has a positive effect on the storage stability of the enzyme. A similar observation was reported by Swarnalatha *et al.* [22] who immobilized α-amylase in Arabic gum. They also considered the high stability of α-amylase after encapsulation of the enzyme in the coating agent. Generally, the combination of coating materials (*i.e.*, Arabic gum and chitosan) with enzyme increases enzyme stability and is the reason for better storage stability of immobilized enzyme, making it suitable for industrial applications.

### 2.5. Effect of Surfactant and Oxidizing Agent on the Stability of Encapsulated Enzyme

**Figure 5:**
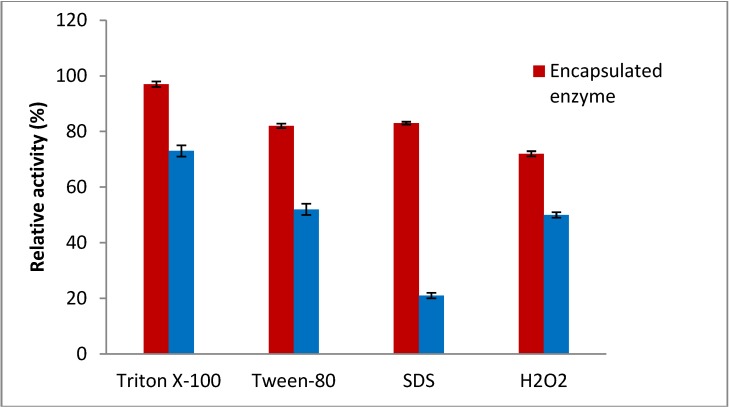
Effect of surfactant and oxidizing agent on enzyme residual activity.

It should be noted that the surfactant could cause loss of the amylase activity and in some cases deactivation of the enzyme activity occurs in the presence of the surfactant and oxidizing agents due to denaturation of the tertiary structure of protein. Surfactants and oxidizing agents are been widely used in various industries and the stability of encapsulated enzyme in the presence of these agents should be studied. Therefore, the effect of non-ionic surfactants (Triton X-100 and Tween-80) and an ionic surfactant (sodium dodecyl sulphate, SDS) as well as an oxidizing agent (H_2_O_2_) on the encapsulated amylase was investigated. The immobilized enzyme was stable in their presence. The encapsulated amylase was highly stable in the presence of the non-ionic surfactants such as Triton X-100 and Tween-80 (Figure 5).

The enzyme also showed great stability in the presence of a strong ionic surfactant (SDS), as it retained 80% of the initial activity when incubated in the presence of 10% (*w/v*) of SDS in the reaction mixture. Moreover, the encapsulated amylase also showed great stability in the presence of oxidising agents (H_2_O_2_) and retained 62% of its initial activity after incubation with 2 M hydrogen peroxide for 1 h (Figure 5). The study revealed that the matrix of Arabic gum-chitosan has low affinity towards the ionic and non-ionic environments caused by different types of surfactants. Such increased stability could be due to immobilization resulting in enhancement of the structural rigidity, which decreases the extent of enzyme distortion upon exposure to surfactants and oxidizing agents [23,24]. The high stability of the encapsulated amylase is one of the most important parameters for the application of enzymes in various industries such as food and detergents.

### 2.6. Particle Size Distribution of the Encapsulated Amylase

**Figure 6:**
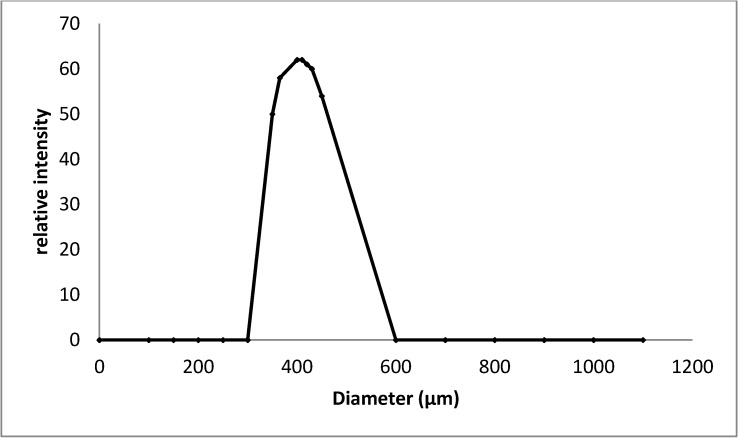
Size distribution curve of encapsulated particles.

Typical dynamic light scattering data on the encapsulated amylase enzyme coated by chitosan and Arabic gum are presented in Figure 6. The particle size distribution was unimodal and the size distribution curve maximum was observed in the range of 400–600 µm. Size of the carriers is considered to be a major parameter in determining the encapsulation efficiency of particulate enzyme delivery. A similar observation was reported by Gassara-Chatti *et al.* [25] who used coating agents for enzyme encapsulation.

### 2.7. Scanning Electron Microscope of Encapsulated Amylase

**Figure 7:**
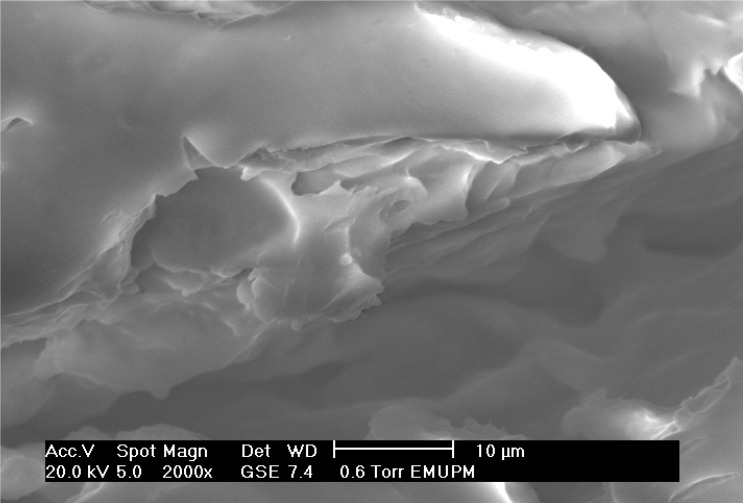
Scanning electron micrograph (SEM) of encapsulated enzyme.

Immobilization of the amylase in Arabic gum-chitosan beads was analyzed by scanning electron microscopy (SEM). Under an electron microscope, the freeze-dried matrix of Arabic gum–chitosan was observed (Figure 7). The SEM result showed that the freeze-dried amylase particles were in a non-crystalline state, had a glass-like structure, amorphous and with a smooth surface. The amorphous glasses produced in the dehydration processes protect the entrapped molecules from opening up due to heat and oxygen exposure.

## 3. Experimental

### 3.1. Chemicals and Plant Material

Red pitaya fruits (*Hylocereus polyrhizus*) were purchased from Passer Brong (Selangor, Malaysia). Ripened fruits free of visual defects were selected based on their size uniformity at the same stage of ripening. The fruits were stored in a cold room at 4 °C until use for the extraction procedure. All chemicals and reagent were of analytical or electrophoresis grade. Sephadex G-200 and DEAE-Sepharose, Bradford Reagent, bovine serum albumin (BSA), Triton X-100, Tween-80 and SDS were obtained from Sigma Chemical Co., (St. Louis, MO, USA). Dibasic sodium phosphate (Na_2_HPO_4_·2H_2_O), monobasic sodium phosphate (NaH_2_PO_4_·H_2_O), sodium acetate, acetic acid, sodium citrate, citric acid, soluble starch, maltose, sodium potassium was obtained from Merck (Darmstadt, Germany).

### 3.2. Preparation of Crude Feedstock

Fresh pitaya fruits (2 kg) were cleaned and rinsed thoroughly with sterile distilled water and dried with tissue paper. The peels were removed and chopped into small pieces (1 cm^2^ each, 1 mm thick); that were quickly blended for 2 min (Model 32BL80 blender, Dynamic Corporation of America, New Hartford, CT, USA) with sodium acetate buffer at pH 5.0 with ratio 4:1, at a temperature of 2.5 °C. The peel-buffer homogenate was filtered through cheesecloth and then the filtrate was centrifuged at 6000 rpm for 5 min at 4 °C and the supernatant (crude enzyme) was collected [26]. The supernatant was kept at 4 °C for use for the purification step using gel filtration and ion exchange chromatography.

### 3.3. Purification of Amylase

A single protein with amylase activity was purified from the red pitaya peel by ammonium sulphate precipitation, gel filtration chromatography on Sephadex G-200 and ion exchange chromatography on a DEAE-Sephadex column. The crude extract was precipitated with 40% ammonium sulphate. The precipitate of each step was dissolved in a small volume of 100 mM sodium acetate buffer (pH 5.0) and dialyzed against the 100 mM sodium acetate buffer (pH 5.0) overnight with frequent (6–8 interval) buffer changes and centrifuged again. The dialyzed solution was subjected to the Sephadex G-200 column, which was eluted with the equilibrating buffer (sodium acetate buffer (pH 5.0) + 0.20 mM NaCl). The flow rate of 1 mL/min was maintained and five fractions of 1.0 mL each were collected. The non-retained fraction from the Sephadex G-200 column was pooled and submitted to one cycle of ion exchange chromatography on DEAE-Sephadex A50. The column was eluted with 100 mM sodium acetate buffer (pH 5.0) to wash the unbound proteins. The bound proteins were eluted with linear salt gradient with 3% NaCl in the same buffer. Finally, the amylase was purified 234.2 fold with a recovery of 72.1% and a specific activity of 3325.6 U/mg protein, respectively.

### 3.4. Microencapsulation Procedure

The partially-purified amylase (50 mg/mL) was mixed with chitosan solution (2%, *w/w*) in 1:1 ratio. The chitosan-amylase mixture was added dropwise into Arabic gum solution (5%, *w/w*) with continuous shaking at 4 °C. Arabic gum was suspended in distilled water at 50 °C and cooled to room temperature. The Arabic gum solution was kept overnight at room temperature to fully hydrate. before addition to the chitosan and amylase mixture [16]. The amylase-chitosan-Arabic gum preparation was filtered using a 50 µm mesh, washed 6 times with 200 mL of buffer (*i.e.*, 50 mM sodium phosphate buffer, pH 7.0) due to remove inorganic contaminants and unbounded coating agents, then, surface dried with absorbent tissue and filter paper. Mixtures were frozen at −40 °C for 24 h before the freeze drying and the frozen samples were lyophilised at −40 °C for 24 h on a VIRTIS Genesis freeze dryer [27].

### 3.5. Amylase Activity Assay and Protein Determination

The amylase activity was measured based on Kammoun *et al.* [28] with slight modifications. The reaction mixture included the enzyme (0.5 mL) and 0.1% of soluble starch (0.5 mL) dissolved in sodium acetate buffer at pH 5.0 (0.1 mL). The mixture, after incubation for 30 min at 70 °C was boiled for 5 min in the presence of 1 mL of DNS. The released reducing sugar was measured by spectrophotometry (BioMate^TM^-3, Thermo Scientific, Alpha Numerix, Webster, NY, USA) at 540 nm by using maltose as standard reducing sugar. One unit of α-amylase activity was defined as the amount of enzyme that produced 1 μmol of maltose per minute under the enzyme activity conditions. Protein concentration was determined by the Bradford [29] method and BSA was used as standard.

### 3.6. Efficiency of Encapsulated Enzyme

The efficiency of the encapsulated amylase was measured by dividing of the enzyme activity after encapsulation to initial enzyme activity multiply to 100% [16].

### 3.7. Storage Stability of Encapsulated Amylase

The encapsulated enzyme was stored for two weeks at 4 °C and then the activity of the enzyme was determined after storage and compared to activity of free enzyme (before encapsulation) using Equation (1).

### 3.8. Effect of Encapsulation on Temperature Stability of Amylase

Temperature stability of encapsulated enzyme and free enzyme was determined by incubating the enzyme in a temperature range of 20 to 100 °C for 1 h. Then the samples were removed and residual amylase activity was determined under standard condition [30].

### 3.9. Effect of Encapsulation on pH Stability of Amylase

The effect of pH on the catalytic activity of amylase, in free as well as encapsulated form, was investigated by measuring initial rates of reaction in buffers with different pH. For amylase, the appropriate pH was obtained using the following buffer solutions: 100 mM sodium acetate buffer (pH 3.0–5.0), 100 mM phosphate buffer (pH 6.0–7.0), 100 mM Tris-HCl buffer pH (7.0–9.0) and 100 mM carbonate (pH 10.0–11.0) [31].

### 3.10. Effect of Encapsulation on Surfactant and Oxidizing Agent Stability of Amylase

The effects of oxidizing agent and surfactant agent on the encapsulated and free amyalse were studied using 2 M H_2_O_2_ as oxidizing agent as well as 5% (*w/w*) Triton X-100, 5% (*w/w*) Tween-80 and 10% (*w/v*) SDS as ionic and non-ionic surfactant agents [32]. Encapsulated enzyme (0.5 mL) was pre-incubated with 0.5 mL of the above additives at 37 °C for 1 h in water batch. Subsequently, 0.5 mL of this reaction mixture was added to 0.5 mL of 0.1% soluble starch as the substrate in 100 mM sodium acetate buffer (pH 5.0) for 30 min at 70 °C to determine the residual enzyme activity. The reaction was stopped by boiling the mixture for 5 min in the presence of 1 mL DNS. Then, the residual enzyme activity was read by spectrophotometry at 540 nm. The procedure as explained above was carried out separately for free enzyme and then the residual enzyme activity of free enzyme in the presence of surfactants and inhibitors was also obtained. Finally, the results of encapsulated enzyme in the presence of the additives were compared with free enzyme under the same conditions.

### 3.11. Particle Size Distribution

Mean particle size and particle size distribution of microencapsulated enzyme were determined by using a dynamic light-scattering particle size analyzer (Mastersizer 2000, Malvern, Worcestershire, UK). To avoid multiple scattering effects, the microencapsulated enzyme was dispersed in deionized water prior to analysis, and then directly placed into the module. A laser beam was directed through the diluted samples, scattered by the sample in a characteristic pattern dependent on their size and detected by an array of photodiodes located behind the cuvette.

### 3.12. Scanning Electron Microscope

The morphology of the encapsulated amylase was performed by Scanning Electron Microscopy (SEM), using a 840A SEM instrument (JEOL, Tokyo, Japan), operated at 20 kV equipped with an INCA 300 EDS analyzer. The dried samples under investigation were placed on sample holders and coated by carbon, using a JEE-4X vacuum evaporator (JEOL, Tokyo, Japan), in order to achieve electrical conductivity.

### 3.13. Experimental Design and Analysis

All the experiments were run using a completely randomised design with three replicates, repeated twice for reproducibility. The analysis of the experimental data with two-way analysis of variance (ANOVA) was conducted followed by the Fisher multiple comparison test at *p* < 0.05. The least significant difference (LSD) test was used to determine if there were significant differences among the samples.

## 4. Conclusions

The encapsulation of the amylase in Arabic gum-chitosan resulted in high encapsulation efficiency. The optimum pH and temperature of the encapsulated enzyme were shifted. The encapsulation process helps to stabilize the enzyme if oxidizing agents and surfactants are present. The study demonstrated that the combination of Arabic gum and chitosan as coating agent protected the amylase enzyme from activity loss during freeze drying. Thus, both coating agents should be considered as potentially important stabilizers for the encapsulation of amylase. The increase in stability and the high activity of the encapsulated amylase could make this approach an attractive choice for biotechnology and enzymology applications.
